# Comparison of red autofluorescing plaque and disclosed plaque—a cross-sectional study

**DOI:** 10.1007/s00784-016-1761-z

**Published:** 2016-03-18

**Authors:** Catherine M. C. Volgenant, Mercedes Fernandez y Mostajo, Nanning A. M. Rosema, Fridus A. van der Weijden, Jacob M. ten Cate, Monique H. van der Veen

**Affiliations:** 1 Department of Preventive Dentistry, Academic Centre for Dentistry Amsterdam (ACTA), University of Amsterdam and VU University, Amsterdam, Gustav Mahlerlaan 3004, 1081 LA Amsterdam The Netherlands; 2 Department of Periodontology, Academic Centre for Dentistry Amsterdam (ACTA), University of Amsterdam and VU University, Amsterdam, The Netherlands

**Keywords:** Dental plaque, Autofluorescence Imaging, Fluorescence, Oral hygiene, Dental plaque index, Dental photography

## Abstract

**Objectives:**

The aim of this cross-sectional study was to assess the correlation between dental plaque scores determined by the measurement of red autofluorescence or by visualization with a two-tone solution. Clinical photographs were used for this study.

**Materials and methods:**

Overnight plaque from the anterior teeth of 48 participants was assessed for red fluorescence on photographs (taken with a QLF-camera) using a modified Quigley & Hein (mQH) index. A two-tone disclosing solution was applied. Total disclosed plaque was clinically assessed using the mQH index. In addition, total and blue disclosed plaque was scored on clinical photographs using the mQH index.

**Results:**

A strong correlation was observed between the total disclosed plaque scored on photographs and the clinical scores (*r* = 0.70 at site level; *r* = 0.88 at subject level). The correlation between red fluorescent plaque and total plaque, as assessed on the photographs, was moderate to strong and significant (*r* = 0.50 at the site level; *r* = 0.70 at the subject level), with the total plaque scores consistently higher than the red fluorescent plaque scores. The correlation between red fluorescent plaque and blue disclosed plaque was weak to moderate and significant (*r* = 0.30 at the site level; *r* = 0.50 at the subject level).

**Conclusions:**

Plaque, as scored on white-light photographs, corresponds well with clinically assessed plaque. A weak to moderate correlation between red fluorescing plaque and total disclosed plaque or blue disclosed plaque was found.

**Clinical relevance:**

What at present is considered to be matured dental plaque, which appears blue following the application of a two-tone disclosing solution, is not in agreement with red fluorescent dental plaque assessment.

## Introduction

Caries and inflammation of the periodontal tissues are the most common oral diseases and are caused by the dental plaque present on the teeth [1]. Dental plaque becomes more pathogenic when present for a longer period on the tooth surface (matured plaque) [2]. Therefore, prevention of oral diseases relies on frequent plaque removal [3].

Several plaque indices have been developed for research purposes to determine the area of the tooth that is covered with plaque. Clinically assessed scores as well as planimetric methods [4] are frequently used after plaque has been disclosed. Often, a two-tone plaque-disclosing solution is used, which supposedly discloses ‘young’ plaque in a pinkish tone and ‘old’ or ‘matured’ plaque in a blueish tone [5, 6]. The pink dye adheres to all plaque, whereas the blue dye adheres and diffuses more easily into the denser/thicker plaque. Hence, the claim of the manufacturer is that young plaque stains pink and matured plaque stains blue-purple.

Dental plaque may also fluoresce red when excited with visible violet light (405 nm) [7, 8]. This fluorescence is an intrinsic characteristic of plaque and is therefore called *auto*fluorescence. However, not all plaque fluoresces red. *In vitro* studies on red autofluorescence from bacteria and biofilms assume that this fluorescence can be attributed to matured or cariogenic plaque [8–10]. While many bacteria that are associated with oral diseases are able to fluoresce red [11–14], modern dietary patterns may affect the autofluorescence of oral bacteria as well. Recent studies on plaque fluorescence indicate that either the volume or the age of the biofilm determines its red fluorescence or that fluorescence is activated by environmental triggers [10, 14, 15]. In a clinical situation, red fluorescent plaque could thus be associated with a thick layer of plaque, maturation of the dental plaque, or the presence of inflamed gingival tissue. And more plaque formation occurs at sites in the oral cavity associated with periodontal inflammation as compared with that in healthy sites [16, 17]. Plaque in some patients may fluoresce within 24 h after professional tooth cleaning, while in others, no red fluorescing plaque is seen after 4 days without oral hygiene. This difference between patients makes it difficult to interpret red fluorescent dental plaque.

A clinical study about dental plaque fluorescence and its correlation with the plaque scores of the total disclosed plaque and the matured plaque portion (blue disclosed portion) could contribute to the understanding of the diagnostic value of red fluorescent plaque.

The aim of this cross-sectional study was to evaluate the correlation between clinically assessed combined (blue and pink) plaque scores and the combined plaque scores assessed on photographs. In addition on photographs, the correlation between scores of dental plaque as made visible by its red autofluorescence or by disclosing with a two-tone solution was assessed. Furthermore, the periodontal condition is known to be a defining factor in the rate of plaque formation [18]. Therefore, the level of gingival health was also assessed clinically, and the bleeding scores per site were compared with the obtained plaque scores to assess a possible relationship between the various methods of plaque assessments and gingival inflammation.

## Materials and methods

### Ethics approval and study participants

This cross-sectional study was performed as part of the baseline assessment of a clinical trial at the periodontology department of the Academic Centre for Dentistry Amsterdam, which was approved by the Medical Ethics Committee of the Academic Medical Centre of Amsterdam (AMC) under registration number NL 37567.018.11. The trial was registered at the Dutch Trial Register under number NTR 3145. The study followed the instructions based on the Declaration of Helsinki (2008).

During the study-entry-assessment period, the participants received oral and written information about the study and could join the study after signing the informed consent. They were subsequently screened for inclusion and exclusion criteria approximately 1 month prior to the start of the study. The participants were considered eligible when they were 18 years of age and in good general health. They needed to have at least six evaluable anterior teeth and no prosthetics or crowns and bridgework in this region and should not have periodontitis as established by the Dutch Periodontal Screening Index (DPSI ≤ 3 minus) [19]. This implies that participants did not have periodontal pockets deeper than 5 mm and had no recessions. Prior to the screening visit, the participants abstained from any means of oral hygiene for at least 12 h. Only participants with a modified Quigley & Hein plaque index score ≥ 2 (clinically assessed) were included to pre-select participants who were able to form overnight dental plaque (‘heavy’ plaque formers). Throughout this study, the modified Quigley & Hein plaque index was used [20]. This is a modification by Turesky et al. [21] in the final description of Paraskevas et al. [22] (mQH index). Participants wearing orthodontic appliances (except for lingual retention wires) or wearing removable (partial) dentures were excluded. Participants were also required to have no untreated cavities at the moment of inclusion, nor restorations with overhanging margins (when clinically assessed with a probe). Additionally, smokers and pregnant women were excluded from participation in this study.

### Clinical procedures

The participants were instructed not to brush the night before the baseline study visit (to develop overnight plaque) and not to eat or drink (except water) 2 h prior to the assessment. At the start of the study visit, the medical history was updated. Fluorescence photographs were taken of the vestibular aspect of the teeth in the upper and lower jaw (cuspid-to-cuspid) in an end-to-end position with a Canon 450-D SLR camera equipped with a Biluminator tube (QLF-D system, Inspektor Research Systems BV, Amsterdam, the Netherlands) to assess the red fluorescence of plaque. Next, the plaque was disclosed using a cotton swab with a two-tone disclosing dye solution (Mira-2-Ton; Hager & Werken, Duisburg, Germany). The disclosing dye solution was applied on a fresh cotton swab till the swab was fully saturated. Subsequently, the swab was gently applied on the tooth surfaces. Excess solution was washed away by allowing the participants to rinse with tap water once. An end-to-end photograph of the teeth after plaque disclosing was captured using an SLR camera with a ring flash (Canon Inc., Tokyo, Japan). Plaque was assessed clinically using the mQH index [22]. Inflammation of the gingiva was assessed using the bleeding on marginal probing index (BOMP) as previously described [23]. BOMP was determined on three upper front teeth and three contra-lateral lower front teeth.

Each clinical parameter was assessed by the same independent calibrated examiners; plaque assessment was performed by G.v.A. and bleeding assessment by E.J.C.M. Clinical parameters were scored from the vestibular aspect of the anterior teeth from the right cuspid to the left cuspid at the upper and lower jaw.

### Photo assessment

The fluorescence photographs and the white-light clinical photographs were renamed with a unique identifier that blinded the examiner to the participant. The vestibular aspect of the anterior teeth (cuspid-to-cuspid, both upper and lower jaw) was assessed. Plaque was visually assessed on the clinical (white light) photographs using the mQH index. This index was applied combining both pink and blue disclosed plaque (*Combi-mQH*) as well as on the portion of blue disclosed plaque (*Blue-mQH*).

**Fig. 1:**
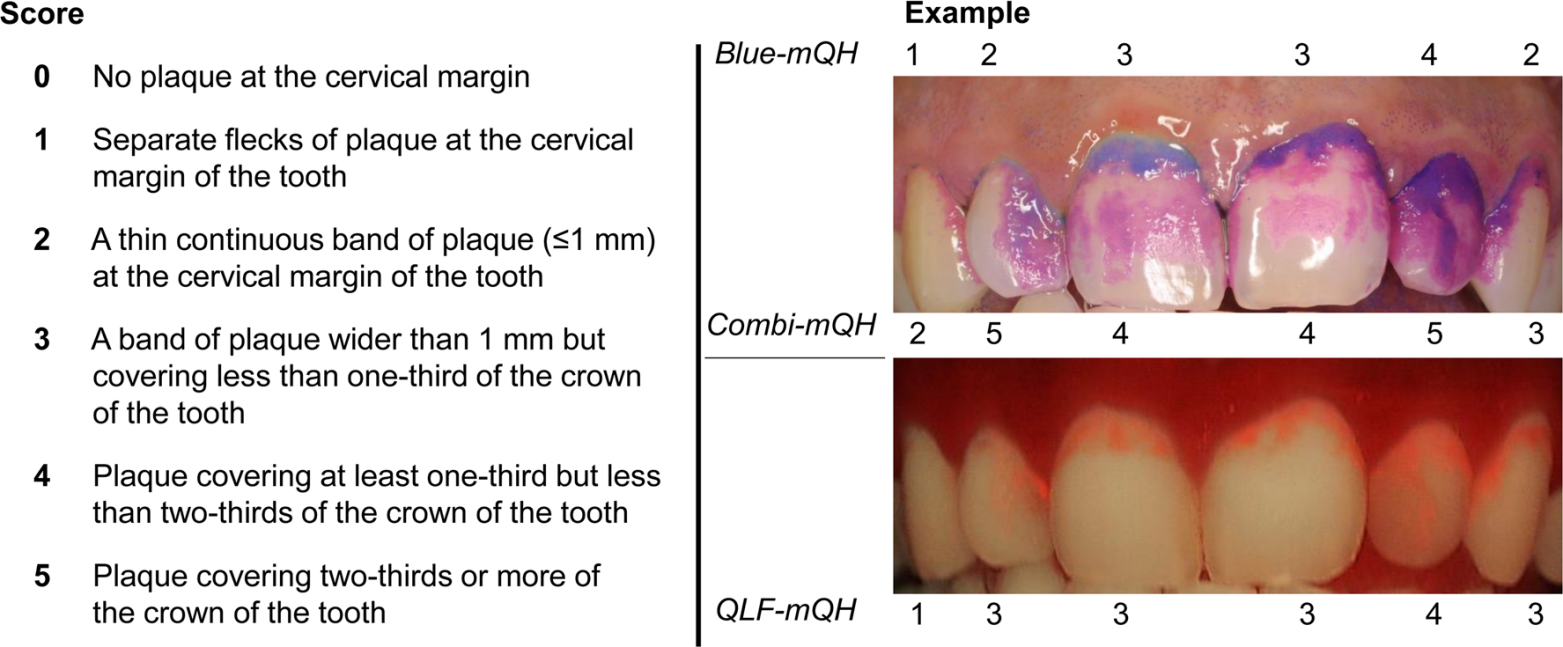
An example of the plaque scoring system is shown for an upper jaw. The modified Quigley & Hein index (*mQH*) scores are described on the left. The buccal surfaces of the anterior teeth are divided into three surfaces (distal-buccal, mid-buccal and mesial buccal). The consensus scores for the mid-buccal surface of each tooth for blue disclosed plaque (*Blue-mQH*) are provided above the white light photograph. The consensus scores for the mid-buccal surface of each tooth for total disclosed plaque (*Combi-mQH*) are provided below the white light photograph. The consensus scores for the mid-buccal surface of each tooth for red fluorescing plaque (*QLF-mQH*) are provided below the fluorescence photograph

Red autofluorescence of dental plaque was visually assessed on the fluorescence photographs. To describe the extent of the red fluorescing plaque, the modified version of the mQH index was used (*QLF-mQH*, Fig. 1).

All photographs were examined twice under similar circumstances by each of the following four independent and calibrated examiners: N.R., M.F., M.V. and C.V. Duplicate assessments of each examiner were performed at least 1 week apart. To analyse and compare plaque indices, consensus scores were derived for red fluorescing plaque, total disclosed plaque and blue disclosed plaque scores.

### Calibration

The four examiners were trained and calibrated using a set of 25 fluorescence photographs and 25 white-light clinical photographs, which were randomly selected from the participants’ screening visit. A training session was organized for the assessment of disclosed plaque using the mQH criteria. Examiner N.R. experienced and calibrated for mQH assessments, trained the other three examiners. An identical procedure was performed for the assessment of red fluorescing plaque using the mQH criteria (Fig. 1). Examiner M.V., experienced in QLF assessments, trained the other three examiners. During the training, each examiner scored the total disclosed plaque, blue disclosed plaque and red fluorescing plaque on 10 photographs. The scores from each examiner were compared and discussed to reach consensus scores. After training, the examiners independently scored the full training set in separate sessions at least 1 week apart.

### Intra-and inter-examiner reliability

The intra-examiner reliability (Cronbach’s alpha) was determined using the first and second assessments of the photographs included in the study. Inter-examiner agreement (Cronbach’s alpha) was determined using the second assessments from each examiner. The intra-examiner consistencies were 0.92–0.99 for the scoring of red fluorescing plaque on the fluorescence photographs, 0.89–0.98 for the total disclosed plaque and 0.55–0.90 for the blue disclosed plaque. The inter-examiner consistencies were 0.45–0.73 for the red fluorescing plaque on the fluorescence photographs, 0.60–0.81 for total disclosed plaque and 0.45–0.70 for the blue disclosed plaque.

### Data analysis

All statistical analyses were performed using SPSS (version 20, IBM Inc. USA). The BOMP scores were dichotomized as score 0 (no bleeding) and score 1 (combined score 1, pinprick bleeding, and score 2, excessive bleeding).

The correlation coefficients at subject level between the different plaque scoring methods were calculated using the Pearson’s *r*. For the site-level comparison, the partial correlation coefficient was calculated to correct for dependencies at subject level. A correlation of 0.1 to 0.3 was considered as a weak positive correlation; a correlation of 0.4 to 0.6 as a moderate positive correlation and a correlation of 0.7 to 0.9 as a strong positive correlation, according to criteria from [35]. To test for differences in plaque scores between bleeding and non-bleeding sites, the Mann-Whitney U test was used. *P* values < 0.05 were considered statistically significant.

## Results

### Participants

Out of 83 volunteers, 32 did not meet the inclusion criteria, and consequently, 51 participants were enrolled in this study. Three participants dropped out after the screening due to other commitments that prevented them from attending the clinic at the baseline appointment. None of these commitments were related to the study. A total of 48 individuals (mean age 22.5 years; range 19–32) participated in the study of which 11 were men (mean age 22.1 years; range 20–26 years) and 37 women (mean age 22.6 years; range 19–32 years). The difference in age between men and women was not significant (*t* = −0.48, *p* > 0.05; independent samples *t* test). The mean clinical mQH index of the 12 anterior teeth of the participants was 2.0 (SD 1.0). The distribution of the DPSI among the participants is displayed in Table 1. The mean level of gingival inflammation as assessed by BOMP in three upper front teeth and three contra-lateral lower front teeth was 0.55 (SD 0.50).

**Table 1:** A summary of the characteristics of the participants and their level of gingival inflammation

	DPSI 0	DPSI 1	DPSI 2	DPSI 3-
Anterior teeth	0 (0 %)	2 (4 %)	44 (92 %)	2 (4 %)
Total mouth	0 (0 %)	2 (4 %)	21 (44 %)	25 (52 %)

DPSI (Dutch Periodontal Screening Index) score 0 stands for a mouth with no pockets deeper than 3 mm, no bleeding on probing and no calculus and/or overhanging restorations present. DPSI score 1 has the same characteristics as score 0, but with bleeding on probing. DPSI score 2 has the same characteristics as score 1, but with calculus and/or overhanging restorations present. DPSI score 3- has pockets of a maximum of 5 mm, bleeding on probing, supra- and subgingival calculus and/or overhanging restorations, but without recessions

### Plaque scoring methods

Table 2 illustrates the correlation of the *Clinical-mQH* with the *Combi-mQH*. As shown in Table 3, the accompanying correlation at site level was high (*r* = 0.70, *p* < 0.001). A high correlation was also found between these two plaque scoring methods at the subject level (*r* = 0.88, *p* < 0.001).

**Table 2:**
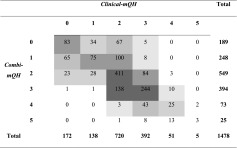
Cross-table visualizing the level of agreement between the clinically assessed total disclosed plaque (*Clinical-*mQH) and the total disclosed plaque assessed by photograph (*Combi-mQH*) at a site level

The *grey boxes* in the table show the value gradients

**Table 3:** Correlation coefficients (at subject level Pearson’s *r* and at site level the partial correlation coefficient) between the different plaque scoring methods *p < 0.001

	*QLF-mQH*		*Combi-mQH*		*Blue-mQH*	
	Subject level	Site level	Subject level	Site level	Subject level	Site level
*Combi-mQH*	0.70*	0.50*	–	–	–	–
*Blue-mQH*	0.50*	0.30*	0.66*	0.39*	–	–
*Clinical mQH*	0.74*	0.48*	0.88*	0.70*	0.56*	0.26*

Table 4a shows a moderate correlation at site level between *QLF-mQH* and *Clinical-mQH* (*r* = 0.48, *p* < 0.001). At a subject level, the correlation between these two values was significant and strong (*r* = 0.74, *p* < 0.001, Table 3). A similar result was found between *QLF-mQH* and *Combi-mQH* at a site level (illustrated in Table 4b, *r* = 0.50, *p* < 0.001) as well as at a subject level (*r* = 0.70, *p* < 0.001). A weak correlation was found between *QLF-mQH* and *Blue-mQH* at a site level (Table 4c, *r* = 0.30, *p* < 0.001) with a moderate correlation at a subject level (*r* = 0.50, *p* < 0.001).

**Table 4:**
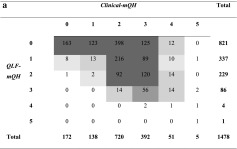

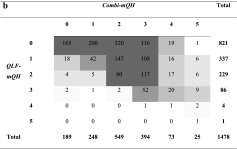

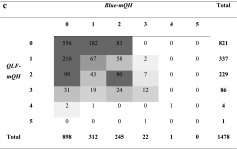
Cross-tables visualizing the level of agreement between red fluorescing plaque (*QLF-mQH*) and the other plaque scoring methods at a site level. The grey boxes in the tables show the value gradients

The correlation between *Blue-mQH* and *Clinical-mQH* was weak at a site level (*r* = 0.26, *p* < 0.001) and moderate at subject level (*r* = 0.56, *p* < 0.001). Similar correlations were found between *Blue-mQH* and *Combi-mQH* (at site level *r* = 0.39, *p* < 0.001; at subject level *r* = 0.66, *p* < 0.001).

All correlation coefficients between the different plaque scoring methods (Table 3) consistently showed numerically greater correlations at a subject level than at a site level.

### Bleeding scores

The Mann-Whitney U test showed that the plaque scores *Clinical-mQH*, *QLF-mQH* and *Combi-mQH* were significantly higher at bleeding sites than at non-bleeding sites (Table 5). For the *Blue-mQH*, no differences were found in blue plaque at bleeding sites compared with blue plaque at non-bleeding sites.

**Table 5:** Bleeding and non-bleeding sites in relation to plaque scores (Mann-Whitney U test)

	Average rank	*Z-score*	
*QLF-mQH*	*no bleeding*	326	−5.54*
	*bleeding*	405	
*Combi-mQH*	*no bleeding*	328	−4.92*
	*bleeding*	403	
*Blue-mQH*	*no bleeding*	374	−0.57♦
	*bleeding*	366	
*Clinical mQH*	*no bleeding*	338	−4.09*
	*bleeding*	398	

*p < 0.001

♦*p* = 0.57

## Discussion

In this study, the plaque scores on photographs showed a strong correlation with the clinical plaque scores of the matching surfaces at both the subject and site levels. This suggests that future clinical studies could use photographs to obtain an indication of the oral hygiene of the anterior teeth and to perform assessments by multiple examiners at a convenient moment, which could enhance the efficiency of a clinical study. The anterior teeth were studied for which it is relatively easy to obtain a good view at the buccal aspect from cuspid to cuspid in the upper and lower jaw with one single photograph. This selection of teeth can provide an indication of the oral hygiene at the full-mouth level.

Between red fluorescent dental plaque and the combined blue and pink disclosed plaque, moderate (site level) to strong (subject level) correlations were found upon clinical assessment and study of photographs. The plaque scores on the QLF-photographs were overall lower when compared with those of the combined blue and pink disclosed plaque, at subject and site levels. The mQH index used for retrieving these plaque scores was originally developed to describe the extent of surface coverage of plaque from the gingiva towards the incisal edges of the teeth and divides the vestibular and lingual surfaces into three areas. The mQH scoring system was applied in this study in a clinical situation. Therefore, a two-tone disclosing agent was used with a presumed discriminatory property to differentiate between ‘new’ and ‘old’ plaque according to the manufacturer. The exact mechanism of action of the dye is not known, but it is reportedly related to the pH and the thickness of the plaque biofilm [5]. The pink component of the dye adheres to all plaque that is present, whereas the blue component adheres and diffuses more easily into denser/thicker plaque, clinically resulting in two distinctive colours of the plaque. This is in contrast with the disclosing agent erythrosine, which stains all present plaque red [24].

A weak (site level) to moderate (subject level) correlation was found between red fluorescent plaque and blue disclosed plaque. Both in the fluorescent plaque scores and in the blue disclosed plaque scores, the high plaque scores (scores 4 and 5) were underrepresented, which suggests that the scores are skewed towards ‘old’ plaque. Although blue-stained plaque is assumed to be older [6, 25], no clear conclusion can be drawn regarding the exact nature of ‘blue’ disclosed plaque. Red fluorescent plaque has been suggested to be matured plaque as well [8–10], but its true nature could not be determined in this study design. However, the observed (weak to moderate) correlations between these two plaque scoring methods were not as expected.

At inflamed sites as indicated by bleeding on marginal probing, higher red fluorescent plaque scores as well as total disclosed plaque scores (both clinical and on photographs) were found. Such an association was not observed between gingival inflammation and the blue disclosed ‘older’ plaque. This seems contradictory to what is generally accepted; dental plaque leads to gingival inflammation when it is present on the tooth surface for a longer period [26]. One determining factor in the development of gingival inflammation is patient susceptibility towards the presence of dental plaque [27, 28]. Furthermore, the assumption that only old plaque causes gingival bleeding does not take the microbiological characteristics of the plaque biofilm into account. Studies relating oral plaque microbiota to gingival inflammation do exist, but these evaluate total plaque or saliva rather than the old and young portions of plaque separately [29, 30]. A recent cross-sectional study in orally healthy participants has reported that the correlation between plaque and bleeding scores on average is low [31]. These results could be explained by the assumption that bleeding on probing most likely represents the impact of the oral hygiene as performed over a longer period of time together with the immunological reaction on this hygiene level rather than being a reflection of the actual oral hygiene status. It can also be argued that blue disclosed plaque does not represent old plaque per se. This is supported by the absence of conclusive literature about the mechanism of action of two-tone dye [5, 6, 25]. Another influencing factor could be the relatively lower intra-examiner consistencies for scoring blue disclosed plaque compared, which indicates that it is more difficult to make an accurate assessment of the blue disclosed plaque.

Environmental factors, such as nutrition, are known to have an effect on autofluorescence of bacteria and of *in vitro* formed biofilms [14, 15]. When gingivitis develops during a period of non-brushing, a change in the oral environment occurs. The association, which was found between gingival inflammation and red fluorescent plaque, could be related to these environmental changes. New studies, preferably in a clinical setting, are needed to determine the relationship between red fluorescence and environmental factors. Although the partial evaluation of the BOMP of the anterior teeth provides an indication of the situation in the whole mouth, future studies should investigate whether similar associations are found when premolar and molar teeth are also included in the assessment of the inflammatory periodontal condition.

Overnight plaque development is representative for people who perform oral hygiene twice daily, resulting in 12 h of plaque accumulation. The participants in the present study were preselected ‘heavy’ plaque formers [32] and were able to form a substantial amount of dental plaque overnight. These specific participants were selected because this study evaluated dental plaque indices for which participants with the full array of plaque scores (0–5) would contribute to a representative assessment of different plaque scoring methods. An adjustment in the exclusion criteria for the participants and a longer period of patient abstinence from oral hygiene habits could have influenced the study results. Similarly, the underrepresentation of sites with high plaque scores (*mQH scores* 4 and 5) could have influenced the study outcome.

The relationship between red fluorescent dental plaque and ‘old’ plaque on natural teeth has not been looked into before. [36] performed a clinical study *on dentures* in which they also used a two tone plaque disclosing solution. They reported smaller red fluorescent plaque coverage compared with the clinical scores of the blue and pink disclosed plaque combined. Their study indicated that red fluorescent plaque is related to matured plaque, although they did not correlate the blue disclosed plaque with the red fluorescent plaque. They observed no correlation between the presence of red fluorescent plaque and the amount of total disclosed plaque area on dentures. This could be the result of other characteristics and the composition of plaque in edentulous patients. They also used an earlier QLF system (the QLF-CLIN system), which was not optimized for detecting red fluorescence. Due to the low contrast in red fluorescence on denture material, and the absence of red fluorescence from the denture material itself, the conclusions of this study are difficult to compare with studies on tooth enamel, such as the present study.

An *in situ* study on bovine enamel [33] reported a correlation between red fluorescent plaque and blue disclosed plaque, although the blue disclosed plaque covered a larger area on the enamel than did the red fluorescent plaque. These results are however difficult to compare with those of the present study because the *in situ* administration of the plaque dyes differs (dipping instead of rinsing). Compared to their fluorescence camera (Vista Proof, Dürr Dental, Germany), the QLF-D camera used in the present study was optimized to better detect red fluorescence. Therefore, the detection level for red fluorescence is lower in the QLF-D system in comparison to the earlier QLF devices and the fluorescence camera use in the studies of [4] and [33], respectively. This may have been one of the reasons for differences between these studies.

Dental plaque forms gradually after cleaning of the tooth surfaces [34]. Future research would therefore preferably comprise a longitudinal study to monitor the changes in the red fluorescence of plaque over time, which would then aid in a complete understanding of the correlation between the fluorescence and the matured plaque. The use of digitized photographs also allows the use of planimetric measurements of dental plaque as well as automatic plaque assessment. These assessments can be easily performed by lay people.

## Conclusion

A strong correlation was found between the clinical plaque scores and the matching surfaces of the plaque scores on photographs. A moderate to strong correlation was found between the portion of red fluorescing plaque and the total disclosed plaque, with total plaque scores that are consistently higher than red fluorescent plaque scores. Red fluorescent plaque and blue disclosed plaque showed similar scores, but the correlation between both was weak to moderate. Higher plaque scores were found at bleeding sites, except for blue disclosed plaque. Because no relationship was found between the blue disclosed ‘old’ plaque and BOMP, the interpretation of blue disclosed plaque as being old plaque can be called into question. Consequently, we can neither confirm nor reject the use of a fluorescence device to screen for matured dental plaque.

## References

[1] Marsh PD (1994). Microbial ecology of dental plaque and its significance in health and disease. Adv Dent Res.

[2] Kolenbrander PE, Palmer RJ Jr, Periasamy S, Jakubovics NS (2010) Oral multispecies biofilm development and the key role of cell-cell distance. Nat Rev Microbiol 8(7):471–480. doi:10.1038/nrmicro2381 10.1038/nrmicro2381 20514044

[3] Axelsson P, Lindhe J (1978). Effect of controlled oral hygiene procedures on caries and periodontal disease in adults. J Clin Periodontol.

[4] Pretty IA, Edgar WM, Smith PW, Higham SM (2005). Quantification of dental plaque in the research environment. J Dent.

[5] Gallagher IH, Fussell SJ, Cutress TW (1977). Mechanism of action of a two-tone plaque disclosing agent. J Periodontol.

[6] Block PL, Lobene RR, Derdivanis JP (1972). A two-tone dye test for dental plaque. J Periodontol.

[7] Heinrich-Weltzien R, Kuhnisch J, van der Veen M, de Josselin de Jong E, Stosser L (2003). Quantitative light-induced fluorescence (QLF)–a potential method for the dental practitioner. Quintessence Int.

[8] Thomas RZ, van der Mei HC, van der Veen MH, de Soet JJ, Huysmans MC (2008). Bacterial composition and red fluorescence of plaque in relation to primary and secondary caries next to composite: an in situ study. Oral Microbiol Immunol.

[9] van der Veen MH, Thomas RZ, Huysmans MC, de Soet JJ (2006). Red autofluorescence of dental plaque bacteria. Caries Res.

[10] Kim Y-S, Lee E-S, Kwon H-K, Kim B-I (2014). Monitoring the maturation process of a dental microcosm biofilm using the quantitative light-induced fluorescence-digital (QLF-D). J Dent.

[11] Coulthwaite L, Pretty IA, Smith PW, Higham SM, Verran J (2006). The microbiological origin of fluorescence observed in plaque on dentures during QLF analysis. Caries Res.

[12] Lennon AM, Buchalla W, Rassner B, Becker K, Attin T (2006). Efficiency of 4 caries excavation methods compared. Oper Dent.

[13] Bjurshammar N, Johannsen A, Buhlin K, Tranæus S, Östman C (2012). On the red fluorescence emission of aggregatibacter actinomycetemcomitans. Open J Stomatol.

[14] Volgenant CMC, van der Veen MH, de Soet JJ, ten Cate JM (2013). Effect of metalloporphyrins on red autofluorescence from oral bacteria. Eur J Oral Sci.

[15] Lee E-S, Kang S-M, Ko H-Y, Kwon H-K, Kim B-I (2013) Association between the cariogenicity of a dental microcosm biofilm and its red fluorescence detected by Quantitative Light-induced Fluorescence-Digital (QLF-D). J Dent 41 (12):1264–1270 10.1016/j.jdent.2013.08.021 24012520

[16] Dahan M, Timmerman MF, Van Winkelhoff AJ, Van der Velden U (2004). The effect of periodontal treatment on the salivary bacterial load and early plaque formation. J Clin Periodontol.

[17] Rowshani B, Timmerman MF, Van der Velden U (2004). Plaque development in relation to the periodontal condition and bacterial load of the saliva. J Clin Periodontol.

[18] Quirynen M, Dekeyser C, van Steenberghe D (1991). The influence of gingival inflammation, tooth type, and timing on the rate of plaque formation. J Periodontol.

[19] Mantilla Gomez S, Danser MM, Sipos PM, Rowshani B, van der Velden U, van der Weijden GA (2001). Tongue coating and salivary bacterial counts in healthy/gingivitis subjects and periodontitis patients. J Clin Periodontol.

[20] Quigley GA, Hein JW (1962). Comparative cleansing efficiency of manual and power brushing. J Am Dent Assoc.

[21] Turesky S, Gilmore ND, Glickman I (1970). Reduced plaque formation by the chloromethyl analogue of victamine C. J Periodontol.

[22] Paraskevas S, Rosema NA, Versteeg P, Timmerman MF, van der Velden U, van der Weijden GA (2007). The additional effect of a dentifrice on the instant efficacy of toothbrushing: a crossover study. J Periodontol.

[23] Van der Weijden GA, Timmerman MF, Nijboer A, Reijerse E, Van der Velden U (1994). Comparison of different approaches to assess bleeding on probing as indicators of gingivitis. J Clin Periodontol.

[24] Paraskevas S, Danser MM, Timmerman MF, Van der Velden U, van der Weijden GA (2005). Optimal rinsing time for intra-oral distribution (spread) of mouthwashes. J Clin Periodontol.

[25] Katayama T, Suzuki T, Okada S (1975). Clinical observation of dental plaque maturation: application of oxidation-reduction indicator dyes. J Periodontol.

[26] Loe H, Theilade E, Jensen SB (1965). Experimental gingivitis in man. J Periodontol.

[27] van der Velden U, Abbas F, Hart AA (1985). Experimental gingivitis in relation to susceptibility to periodontal disease. (I.) clinical observations. J Clin Periodontol.

[28] Fransson C, Mooney J, Kinane DF, Berglundh T (1999). Differences in the inflammatory response in young and old human subjects during the course of experimental gingivitis. J Clin Periodontol.

[29] Lie MA, Danser MM, van der Weijden GA, Timmerman MF, de Graaff J, van der Velden U (1995). Oral microbiota in subjects with a weak or strong response in experimental gingivitis. J Clin Periodontol.

[30] Huang S, Li R, Zeng X, He T, Zhao H, Chang A, Bo C, Chen J, Yang F, Knight R, Liu J, Davis C, Xu J (2014). Predictive modeling of gingivitis severity and susceptibility via oral microbiota. ISME J.

[31] Oliveira SC, Slot DE, Celeste RK, Abegg C, Keijser BJ, Van der Weijden FA (2015) Correlations between two different methods to score bleeding and the relationship with plaque in systemically healthy young adults. Journal of clinical periodontology 42 (10):908–913 10.1111/jcpe.12435 26212602

[32] Simonsson T, Ronstrom A, Rundegren J, Birkhed D (1987). Rate of plaque formation–some clinical and biochemical characteristics of “heavy” and “light” plaque formers. Scand J Dent Res.

[33] Raggio DP, Braga MM, Rodrigues JA, Freitas PM, Imparato JC, Mendes FM (2010). Reliability and discriminatory power of methods for dental plaque quantification. J Appl Oral Sci.

[34] Quirynen M, van Steenberghe D (1989). Is early plaque growth rate constant with time?. J Clin Periodontol.

[35] Dancey C, Reidy J (2004). Statistics without maths for psychology: using SPSS for windows.

[36] Coulthwaite L, Pretty IA, Smith PW, Higham SM, Verran J (2009). QLF is not readily suitable for in vivo denture plaque assessment. J Dent.

